# Neonatal Procalcitonin Intervention Study (NeoPInS): Effect of Procalcitonin-guided decision making on Duration of antibiotic Therapy in suspected neonatal early-onset Sepsis: A multi-centre randomized superiority and non-inferiority Intervention Study

**DOI:** 10.1186/1471-2431-10-89

**Published:** 2010-12-08

**Authors:** Martin Stocker, Wim CJ Hop, Annemarie MC van Rossum

**Affiliations:** 1 PICU Royal Brompton Hospital, Sydney Street, SW3 6NP London, UK; 2 Dept of Biostatistics, Erasmus Medical Centre, 3015 GJ Rotterdam, NL; 3 Erasmus Medical Centre-Sophia Children's Hospital, 3015 GJ Rotterdam, NL

## Abstract

**Background:**

Early diagnosis and treatment of the newborn infant with suspected sepsis are essential to prevent severe and life threatening complications. Diagnosis of neonatal sepsis is difficult because of the variable and nonspecific clinical presentation. Therefore, many newborns with nonspecific symptoms are started on antibiotic treatment before the presence of sepsis has been proven. With our recently published single-centre intervention study we were able to show that Procalcitonin determinations allowed to shorten the duration of antibiotic therapy in newborns with suspected early-onset sepsis.

**Methods/Design:**

The study is designed as randomized controlled international multicenter intervention trial on the efficacy and safety of Procalcitonin guided treatment. Term and near-term infants (gestational age ≥ 34 0/7 weeks) with suspected sepsis in the first 3 days of life requiring empiric antibiotic therapy will be included. The duration of antibiotic therapy in the standard group is based on the attending physician's assessment of the likelihood of infection (infection unlikely, possible, probable or proven). In the Procalcitonin group, if infection is considered to be unlikely or possible, antibiotic therapy is discontinued when two consecutive Procalcitonin values are within the normal range. Co-primary outcome measures are the duration of antibiotic therapy (superiority aspect of the trial) and the proportion of infants with a recurrence of infection requiring additional courses of antibiotic therapy and/or death in the first month of life (safety of study intervention, non-inferiority aspect of the trial). The number of infants to be included equals 800 per arm. With these numbers the power of the study to demonstrate superiority for duration of antibiotic therapy as well as non-inferiority regarding safety, i.e. excluding a disadvantage difference larger than 2% for the experimental arm, will both be greater than 80%.

**Discussion:**

Benefit of the study is a possible limitation of unnecessary use of antibiotics. The results of our first study suggest that there is a low risk on discontinuing antibiotic treatment too early, resulting in the development of a neonatal infection with its morbidity and mortality.

**Trial registration:**

This trial is registered in the U.S. National Institutes of Health's register, located at http://www.clinicaltrials.gov. (NCT00854932).

## Background

Infections are the single largest cause of neonatal deaths globally [1]. Based on the onset, neonatal sepsis is classified into two major categories: early onset sepsis, which usually presents with respiratory distress within 72 hours of age and late onset sepsis that usually presents with septicemia after 72 hours of age. Sepsis in neonates is a significant contributor to morbidity and death, with mortality rates varying from 3% to as high as 50% in some series, especially with gram-negative pathogens [2-6]. The incidence of early-onset sepsis in term neonates in The Netherlands 2003-2006 is approximately 0.6% [7].

Early diagnosis and treatment of the newborn infant with suspected sepsis are essential to prevent severe and life threatening complications. In contrast to the clear and valuable therapeutic options, the diagnosis of suspected early-onset neonatal sepsis is challenging. The early signs of sepsis in the newborn are non-specific. Therefore, many newborns with nonspecific symptoms undergo diagnostic studies and the initiation of treatment before the presence of sepsis has been proven. Blood culture is currently the gold standard for the diagnosis of sepsis. However, in addition to the fact that culture reports are available only after 48-72 hours, blood cultures are frequently false negative due to the small amount of blood that can be drawn from neonates [8]. The reliability of most laboratory markers, including white blood cell count (WBC), C-reactive protein (CRP), Procalcitonin (PCT) and IL-6 for the diagnosis of neonatal infection has been assessed in highly diverse groups of ill neonates with a mixture of diagnoses and conditions and has yielded variable results [9]. If culture results come back negative after 48-72 hours, the clinician has to decide whether to provide continued treatment.

In the era of multidrug resistance, it is mandatory to avoid unnecessary use of antibiotics to treat non-infected infants. In addition, the intravenously administration of antibiotics necessitates admission of the neonate to the hospital and thereby separation of mother and child in this delicate period of life. Thus rapid diagnostic test(s) that differentiate infected from non-infected infants, particularly in the early newborn period, have the potential to make a significant impact on neonatal care. In an effort to reduce the use of antimicrobial agents in neonates, clinical studies have been undertaken using the biomarker CRP to safely influence the length of antimicrobial therapy [10,11]. Thus far, no evidence has been presented that using CRP can make an impact on the length of antimicrobial therapy.

Another biomarker that has been discovered more recently, PCT, is proven to be a good marker of severe, invasive bacterial infections in children. All studies on severe, invasive bacterial infections in children report higher sensitivities and specificities of PCT than for CRP [12-16]. PCT is a 116-aminoacid peptide and one of the precursors of calcitonin. The physiological function of calcitonin remains unknown. No disorders attributable to either an excess or a deficiency of calcitonin have been identified. Most microbial infections induce a ubiquitous increase in CALC1 gene expression and a subsequent release of calcitonin precursors from all tissues and cell types throughout the body [17]. In bacterial infections, PCT increases from concentrations in the picogram range (below the detection level of current PCT assays) to plasma concentrations ranging from 1 to 1000 ng/ml. This increase often correlates with the severity of the disease and with mortality [18-21]. Increases in PCT occur more rapidly than increases in CRP. PCT can be detected in the plasma 2 hours after the injection of endotoxins. Within 6-8 hours, PCT concentrations rise and a plateau is reached after approximately 12 hours [22]. CRP can be detected in the plasma after 12 h and reaches a plateau after 20-72 hours. PCT and CRP decrease to their normal values after 2-3 days and 3-7 days, respectively [23-25].

The use of PCT as a marker of neonatal bacterial infection is complicated by several factors. First, infants with respiratory distress syndrome, hemodynamic failure, perinatal asphyxia, intracranial hemorrhage, pneumothorax, or after resuscitation have raised serum PCT concentrations that do not differ from those of septic neonates up to 48 h after onset of clinical signs of distress or infection [26-28]. Second, a physiological increase of PCT has been reported up to 48 h post partum [29]. Third, prepartum and intrapartum administration of antibiotics may affect PCT concentrations in the umbilical cord [30], and postnatal administration of antibiotics will decrease PCT concentrations more rapidly than CRP concentrations [31,32]. When these pitfalls are taken into account, PCT performs better than CRP in diagnosing neonatal bacterial infection.

Chiesa et al developed a nomogram for PCT [33] and Assumma and colleagues performed a longitudinal study on PCT values in healthy neonates [34]. In his findings Chiesa was able to report two major differences in between healthy and septic neonates which formed the basis of his nomogram. Firstly, the level of elevation in PCT was much higher in septic neonates versus healthy newborns and secondly, the absence of a decrease of PCT values after the initial cytokine release post-partum is indicative of a bacterial infection. With all PCT values being increased during the first two days of life, a reference range covering this time period with intervals of several hours is a tool to identify septic neonates. The adult reference ranges apply from three days after birth. Using PCT in this manner has been proven extensively to be a very reliable marker for the diagnosis of neonatal sepsis [35-39].

In recent years a novel indication for the use of PCT has been discovered, related to its described high negative value. It has been reported in many interventional trials [40-44] that a low PCT indicates the absence of a need for antimicrobial therapy. In several countries the recent adult intensive care guidelines have been altered to the extent that PCT has displaced CRP in the recommendations [45,46]. Applying this principle to neonatology, we performed a single-centre intervention trial in Lucerne, Switzerland that showed that serial PCT determinations allowed to shorten the duration of antibiotic therapy in term and near-term infants with suspected early-onset sepsis [47]. This study is designed to test the reliability of a PCT-based strategy in a larger cohort of neonates.

## Methods/Design

The purpose of this trial is to evaluate whether PCT measurements are able to reduce antibiotic usage in suspected neonatal early-onset sepsis by reducing the duration of antibiotic treatment with unchanged outcome. The study is designed as randomized open controlled international multicenter intervention trial on the efficacy (superiority aspect) and safety (non-inferiority aspect) of PCT guided treatment.

### Outcome measures

Co-primary outcome measures are the duration of antibiotic therapy and the proportion of infants with a recurrence of infection requiring additional courses of antibiotic therapy (within 72 hours after ending antibiotic therapy) and/or death in the first month of life (safety of study intervention). A secondary outcome measure is the length of hospitalisation stay.

### Inclusion/Exclusion criteria

Inclusion criteria: Term and near-term infants with a gestational age ≥ 34 0/7 weeks, age 0-3 days of life, suspected sepsis in the first 3 days requiring empiric antibiotic therapy, parental consent. Exclusion criteria are severe congenital malformations and surgical procedures before or during the study. In case of surgical procedures during this trial patients will be excluded from the study and its analyses. These children will be treated by current protocols of care, including protocols for antibiotic therapy. Patients are not allowed to enter the trial for a 2^nd ^time.

Patients will be randomized between t = 0 and t = 12 h after the initiation of antibiotic treatment. Part of the neonates will be eligible for inclusion immediately after birth. Since it is not always possible to ask informed consent from parents immediately after a child is born, this timeframe for randomization of 12 hours is necessary. Randomization will be to either a standard treatment based on conventional laboratory parameters (standard group) or to PCT-guided treatment (PCT group) blocked by centre: Randomization is done by drawing group assignment cards in opaque sealed envelopes (Switzerland) and by computer based digital randomization (The Netherlands).

### Statistical analyses

This trial is designed to exclude a difference in the rates of re-infection or death greater than 2% (non-inferiority aspect of the trial). Assuming a 2% reinfection/death rate in each group, 770 patients are required per arm for a power of 80% at one-sided alpha of 0.025. Based on our data of the study in Lucerne [47], with this number of patients a difference between mean antibiotic therapy durations of 10 hours can be detected at two-sided alpha of 0.05 with a power of 95% (superiority aspect of the trial). To allow for some unevaluable cases 800 per group will be included.

Primary analyses: The two-sided 95% confidence interval for the difference (experimental - control) in re-infection/death rates will be calculated for the total randomized arms and if this difference excludes +2%, non-inferiority is considered to be shown [48]. Comparison of durations of antibiotic therapy will be done using the Mann-Whitney test with stratification by centre. Any patients dying will be considered as the worst outcome in this evaluation and their duration will be set at the highest duration found. All analyses will be done according to the intention-to-treat principle and will be done with stratification by centre. A per-protocol analysis, excluding patients with major protocol violations, will also be done.

Secondary analysis: Exploratory analysis of primary endpoints regarding the relations with and clinical outcomes assessed at T = 24-72 h will be done using logistic regression or Anova. In the Anova the duration's antibiotic treatment will be transformed logarithmically. All tests are two-sided and p = 0.05 is the limit of significance. These analyses will be informal only because it cannot be excluded that the ratings of clinical outcomes at T = 24-72 h are influenced by the PCT results in the PCT-group. In a secondary analysis reinfection/deaths rates will be compared between treatment arms using random effects logistic regression allowing for centre as a random factor.

### Laboratory examinations

Complete blood counts and CRP concentrations are obtained in all patients. Serial PCT measurements are performed in all patients of the PCT group and if blinding for the PCT results of the standard group is feasible also in the standard group. Laboratory examinations are stopped by ending of antibiotic therapy. Blood sampling will be limited to normal frequencies already used in daily practice for neonatal care. This means that for this trial blood will collected at time points 0 (moment of inclusion and start point of antibiotic therapy), 24 h (+/- 6 h) after inclusion, between 36 to 72 h after inclusion and then 24-48 hourly until end of antibiotic therapy. One additional sample will be collected in the PCT group 12 h (+/- 6 h) after inclusion. With the exception of the one additional sample in the PCT group, no additional punctures will be done for research purposes.

Procalcitonin will be measured on the automated Kryptor platform, supplied by the firm BRAHMS AG of Hennigsdorf, Germany, via the Roche Elecsys BRAHMS procalcitonin assay. The BRAHMS Kryptor sensitive procalcitonin will be applied on this platform using Time Resolved Amplified Cryptate Emission (TRACE) technology. This assay is based on a polyclonal antibody against calcitonin and a monoclonal antibody against katacalcin, which binds to the calcitonin and katacalcin sequence of the calcitonin prohormone. The test is considered a homogeneous immunoassay (sandwich principle) and is validated on serum and plasma (EDTA and heparin) matrix. The direct measuring range of the assay is from 0.02-50 ng/ml, with automated dilution extending the upper range to 1.000 ng/ml. The Functional Assay Sensitivity (FAS) is 0.06 ng/ml. Procedure time of the assay is very short at 19 minutes. The needed sample volume is limited to 50 micro litres. The Roche Elecsys BRAHMS procalcitonin assay equally uses an immunoassay based on a sandwich principle based on a polyclonal antibody against calcitonin and a monoclonal antibody against katacalcin, which binds to the calcitonin and katacalcin sequence of the calcitonin prohormone. The direct measuring range of the assay is from 0.02-100 ng/ml. The Functional Assay Sensitivity (FAS) is 0.06 ng/ml. Procedure time of the assay is also very short at 18 minutes. The needed sample volume is limited to 30 micro litres. The assay is validated on serum and plasma (EDTA and heparin) matrix. CRP and all other requested laboratory assays will be measured on the routine analyzers of the various sites and will be made available to the physician through the routine laboratory systems in place.

### Procalcitonin-guided decision making

**Figure 1:**
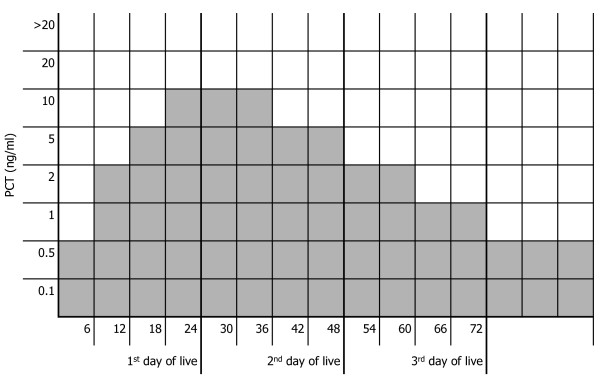
**Normal age-adapted PCT ranges. Grey boxes = age-adapted normal range **[47].

The normal age-adapted PCT ranges according to our previous study are shown in Figure 1. To provide a margin of safety, the maximal normal value of PCT is defined as 10 ng/ml (18-36 hours of life) which, according to the literature is about 50% of the highest PCT concentrations measured in neonates with respiratory distress not related to infection [33].

At time-point T = 24-72 h (one time-point between 24-72 h after start of antibiotic therapy: time-point of positive culture result or time-point of early stop of antibiotic therapy (less than 72 h of therapy) or 72 h after start of therapy) neonates will be assessed and divided into categories of risk classification (Table 1 and 2): infection proven (category 1), infection probable (category 2), infection possible (category 3), and infection unlikely (category 4). The treating physician will decide in which risk classification a neonate belongs. Expected distribution based on the results of our first study [47]: Category 1 50%, Category 2 30-35%, Category 3 and 4 15-20%.

**Table 1:** Risk of infection classification categories

Category 4: Low risk (Infection unlikely)	**One **abnormal finding:
	- pre/perinatal risk factor*, OR
	- suspect clinical symptom*, OR
	- abnormal laboratory examination*
**Category 3:** **Intermediate risk** (Infection possible)	**Two **abnormal findings:
	- pre/perinatal risk factors*, AND/OR
	- suspect clinical symptoms*, AND/OR
	- abnormal laboratory examinations*

**Category 2:** **High risk** (Infection probable)	**Three **abnormal findings:
	- pre/perinatal risk factors*, AND/OR
	- suspect clinical symptoms*, AND/OR
	- abnormal conventional laboratory examinations*

**Category 1:** **Proven infection**	≥ 1 abnormal finding (pre/perinatal risk factors, clinical symptoms, laboratory examinations) AND a **positive culture**

The assessment of risk of infection classification is independent of serum PCT levels.

* See assessment guidelines (Table 2)

**Table 2:** Assessment guidelines

Pre/perinatal risk factors	Mother group B streptococcus (GBS) positive
	Maternal prolonged rupture of membranes (PROM) > 18 h
	Maternal chorioamnionitis (fever >38.5°, fetal tachycardia)
	Birth before 35 weeks of gestational age
**6 groups of suspect clinical signs and symptoms**	Respiratory distress/apnea
(signs and symptoms of one group = one abnormal finding)	Tachycardia/bradycardia
	Arterial hypotension/poor perfusion
	Hypothermia/hyperthermia
	Seizure/floppy infant/irritability/lethargy/poor feeding
	Vomiting/feeding intolerance/ileus

**Conventional laboratory examinations**	White blood cell count < 5 G/L
	CRP > 10 mg/L

The duration of antibiotic therapy in the standard group is based on the attending physician's assessment of the risk classification during hospitalisation: in category 4 patients, antibiotics are given for 2 - 3 days, in category 3 patients for 5 - 7 days. In the PCT group, if infection is considered to be unlikely or possible, antibiotic therapy is discontinued when two consecutive PCT values are within the normal range (Figure 1 Table 3). Antibiotic therapy can be continued despite fulfilled PCT criteria at the discretion of the attending physician. These diversions from the stopping rules will be reported for further analysis. If infection is proven (category 1) or considered to be probable (category 2) antibiotics are given for 7 - 21 days in both groups.

**Table 3:** Duration of antibiotic therapy

	Standard group	PCT group
**Category 1 and 2**	7 - 21 days	7 - 21 days

**Category 3**	5 - 7 days	at least 24 hours, stop after 2 consecutively negative PCT-values

**Category 4**	36 - 72 hours	at least 24 hours, stop after 2 consecutively negative PCT-values

### Follow up

Patients with a recurrent infection will not enter the study for a second time. Recurrent infections will be analysed for focus and relapse of previous infection and will be treated at the physician's discretion. Recurrent infections in this trial will be defined as a new infection occurring within 72 hours after stopping antibiotic therapy for the initial infection. Any recurrent infection will be reported immediately to the steering committee.

Follow-up of the patients will be performed as in standard practice. Parents of discharged patients have 24 hours/day, 7 days/week access to the hospital and to contact a paediatrician on call. Phone numbers to contact the department will be provided upon discharge of the child. A follow-up interview after 1 month will be done with questions about undercurrent illness, physician visits, medications and hospitalisations.

### Adverse events

All serious adverse events will be reported to the principal investigator within 24 hours after their occurrence. Also the data and safety monitoring board and the ethical committee that approved the protocol will be informed by the principal investigator, according to the requirement of the ethical committee. All adverse events will be followed until they have abated, or until a stable situation has been reached. The members of the data and safety monitoring board will be blinded to the assigned intervention arm of the patient. At any time the members of this board can ask to be unblinded by requesting the treatment code from the independent statistical centre.

### Data collection and management

All data are collected in the neonatal intensive care department or pediatric ward. The medical history and used medication can be obtained from the patient's medical record. All collected data will be stored anonymously in the study database. Data management will be performed by the local investigator or the trial nurses of the participating center. The subjects will be identified by a trial identification number. Data base integrity and data safety as well as privacy are warranted by the participating research hospitals. Source data verification will be conducted by an independent Data Monitor.

An independent data- and safety monitoring committee is responsible for the ongoing conduct of the study, based on unbiased and independent review of efficacy and safety data. Dr. R. Oostenbrink, paediatrician and clinical epidemiologist, Erasmus MC-Sophia, Rotterdam and Prof. E. Lesaffre, statistician at Erasmus MC will serve as members from The Netherlands and Prof. G. Schubiger, neonatologist and head of the local ethical committee in Lucerne and Dr. C. Hagmann, consultant neonatologist at the University Hospital in Zurich from Switzerland. The committee assesses the progress of the trial, and the safety data and advises whether to continue, modify or stop the trial every 3 months. The DMC charter informs about purpose, composition, responsibilities and structure of the committee. The board will operate under strict confidentiality.

### Ethical approval and trial registration

For this trial a nationwide ethical approval was requested for The Netherlands and local ethical approval was requested for all participating centres by their local ethical committee. Further participating study centers outside Switzerland and The Netherlands have to request approval by their ethical committee and to present the approval to the steering committee of the study. This trial will be conducted in accordance with the ethical guidelines of the World Medical Association's declaration of Helsinki Ethical Principles for Medical Research Involving Human Subjects as adopted by the 18th WMA General Assembly, Helsinki, Finland, June 1964, and amended by the 29th WMA General Assembly, Tokyo, Japan, October 1975; 35th WMA General Assembly, Venice, Italy, October 1983; 41st WMA General Assembly, Hong Kong, September 1989; 48th WMA General Assembly, Somerset West, Republic of South Africa, October 1996; 52nd WMA General Assembly, Edinburgh, Scotland, October 2000; 53th WMA General Assembly, Washington 2002 (Note of Clarification on paragraph 29 added); 55th WMA General Assembly, Tokyo 2004 (Note of Clarification on Paragraph 30 added); 59th WMA General Assembly, Seoul, October 2008. Also, this trial will comply with the regulations set forth by the Medical Research Involving Human Subjects Act (WMO) and other guidelines, regulations and Acts that apply.

This trial is registered in the U.S. National Institutes of Health's register, located at http://www.clinicaltrials.gov. under number NCT00854932.

## Discussion

It is essential for observational studies to compare new markers with the gold standard. The gold standard for the diagnosis of neonatal early-onset sepsis, i.e. a positive blood culture is often unreliable because of the frequent use of intrapartum antibiotics or insufficient amounts of blood available for culture [8]. Therefore, the problem of all observational studies of neonatal early-onset sepsis is the definition of sepsis. An intervention study offers a valuable alternative because the calculation of the main results is independent of the definition of sepsis. A control group is necessary to demonstrate that results from the intervention group differ from what will be observed in the standard group. We choose the design of a non-inferiority trial to show that a reduced duration of antibiotic therapy (superiority aspect) doesn't change the outcome (recurrence of infection, mortality).

As this trial will focus on the effectiveness and safety of a Procalcitonin-guided antibiotic treatment, it will not focus on the clinical reasons why a clinician will decide to start antibiotics. The start of antibiotic treatment is solely the decision of the attending neonatologist. The purpose of the choice to not interact with this clinical decision is to evaluate current clinical practice the closest without creating a bias towards biomarker started (or withheld) therapy. Therefore, when designing this intervention study, the definition of sepsis is less crucial than in an observation study, because in an intervention study all newborns are suspected to have bacterial infection and are therefore treated with antibiotics. The key point is the comparison of the outcomes after intervention. The probability of infection must be assessed by the attending physician at t = 24-72 h during the hospitalisation, because antibiotic treatment is not discontinued in neonates with proven or a high risk of infection based on maternal risk factors, clinical signs and symptoms, and conventional laboratory parameters.

Benefit of the study is a possible limitation of unnecessary use of antibiotics. On a population level, unnecessary long-term use of broad-spectrum antibiotics is a serious concern because it can promote the development of resistant bacteria, which will result in untreatable infections over time. Because the treatment consists of intravenously administered antibiotics, this means admission to the hospital for the neonate with separation of mother and child during these important first days of life. It is obvious that shortening this period when possible is desirable. It will also result in fewer painful punctures. Neonates need punctures for new intravenous lines very frequently, so shorter treatment will result in fewer punctures.

The burden of the trial is minimal, because only one extra time point for blood drawing will be done. For the other time points no additional diagnostic procedures are needed. The additional burden consists of a couple of extra blood drops during routine blood sampling.

The estimated risk is low. There is a low risk on discontinuing antibiotic treatment too early, resulting in the development of a neonatal infection with its morbidity and mortality. Based on follow-up data of our first study no mortality was observed in 121 neonates [47]. In only two children antibiotic treatment was restarted with good outcome and without proof for secondary infections due to early stop of primary antibiotic therapy.

## Competing interests

The authors declare that they have no competing interests. BRAHMS diagnostica, manufacturer of the procalcitonin assay provided the testing kits for PCT determinations but is not involved in any other aspect of the study or manuscript preparation. No financial compensation is offered to participating patients.

## Authors' contributions

MS is principal investigator of the study and is responsible for the design of the study, for coordination, supervision and data management in Switzerland and Prague. WH is participated in the design of the study, performs the statistical analysis and is responsible for future data analysis. AR is Co-principal investigator and is responsible for the design of the study, for coordination, supervision and data management in The Netherlands. All authors read and approved the final manuscript.

## Pre-publication history

The pre-publication history for this paper can be accessed here:

http://www.biomedcentral.com/1471-2431/10/89/prepub
